# Vulnerability and “minor” developmental disorders in late preterm infants

**DOI:** 10.1186/1824-7288-40-S2-A21

**Published:** 2014-10-09

**Authors:** Giovanni Cioni, Francesca Tinelli

**Affiliations:** 1 Department of Developmental Neuroscience, Stella Maris Scientific Institute, Calambrone, 56128-Pisa, Italy; 2 Department of Clinical and Experimental Medicine, University of Pisa, Pisa, Italy

## 

The National Institute of Child Health and Human Development panel reviewed the evidence of increased risk of infants with a gestational age of 34-36 weeks and, in 2006, changed the earlier definition of “near term” to “late preterm (LPT)”. LPT infants represent 70% of all the whole population of preterm but while it is known that they are at major risk of mortality and morbidity than term infants, less is known about their development outcome. Samra et al. [1] in 2011 published a review about this topic based on 817 articles but their conclusion was that, due to paucity and heterogeneity of the existing data, there was no clear characterization of the long-term risks. Since then some other interesting papers have been published, quite all in the direction that LPT children have some “minor” problems. In 2013, for example, Vohr B. [2] suggested that LPT infants are at increased risk of neurologic impairments, developmental disabilities, school failure, and behavior and psychiatric problems suggesting also that for each 1 week decrease in gestational age below 39 weeks, there are stepwise increases in adverse outcomes after adjusting for confounders. In 2014, Chan et al. [3] described the negative impact of LPT birth on academic outcomes at 7 years and Brumbaugh et al. [4] the negative impact on executive function at preschool age.

A possible explanation of these results is i) the demonstrated major vulnerability to the brain injury in the late preterm infant respect to the term infant, particularly involving the white matter [5] since that at 34 weeks the late preterm brain weights only 65% of the term brain and ii) the possible role of the extrauterine life compared with the intrauterine life during the last weeks of gestation.
